# Experiences of case managers in providing person-centered and integrated care based on the Chronic Care Model: A qualitative study on embrace

**DOI:** 10.1371/journal.pone.0207109

**Published:** 2018-11-15

**Authors:** Ronald J. Uittenbroek, Sijrike F. van der Mei, Karin Slotman, Sijmen A. Reijneveld, Klaske Wynia

**Affiliations:** 1 Department of Health Sciences, Community & Occupational Medicine, University Medical Center Groningen, University of Groningen, Groningen, the Netherlands; 2 Department of Health Sciences, Applied Health Research University Medical Center Groningen, University of Groningen, Groningen, the Netherlands; 3 Department of Neurology University Medical Center Groningen, University of Groningen, Groningen, the Netherlands; Maastricht University CAPHRI Care and Public Health Research Institute, NETHERLANDS

## Abstract

**Background:**

Due to the rise in the number of older adults within the population, healthcare demands are changing drastically, all while healthcare expenditure continues to grow. Person-centered and integrated-care models are used to support the redesigning the provision of care and support. Little is known, however, about how redesigning healthcare delivery affects the professionals involved.

**Objectives:**

To explore how district nurses and social workers experience their new professional roles as case managers within Embrace, a person-centered and integrated-care service for community-living older adults.

**Methods:**

We performed a qualitative study consisting of in-depth interviews with case managers (district nurses, n = 6; social workers, n = 5), using a topic-based interview guide. Audiotaped interviews were transcribed verbatim and analyzed using qualitative content analysis.

**Results:**

The experiences of the case managers involved four major themes: 1) the changing relationship with older adults, 2) establishing the case-manager role, 3) the case manager’s toolkit, and 4) the benefits of case management. Within these four themes, subthemes addressed the shift to a person-centered approach, building a relationship of trust, the process of case management, knowledge and experience, competencies of and requirements for case managers, and the differences in professional background.

**Discussion:**

We found that this major change in role was experienced as a learning process, one that provided opportunities for personal and professional growth. Case managers felt that they were able to make a difference, and found their new roles satisfying and challenging, although stressful at times. Ongoing training and support were found to be a prerequisite in helping to shift the focus towards person-centered and integrated care.

## Introduction

Modern societies are challenged by an increase in life expectancy and the consequent rise in the number of older adults within the population. Healthcare demands are changing drastically, while healthcare expenditure continues to grow.[1] Contemporary healthcare systems, once designed to solve single, acute, and short-term diseases, need to be transformed in order to be able to meet the changing demands while lowering cost. Person-centered and integrated-care models are used to support the redesigning the provision of care and support,[2–4] and new professional roles are being introduced for this purpose. However, little is known about how these professionals experience their new roles.[5]

Case management plays a central role in most person-centered and integrated care services. In case management, an individual or a small team is responsible for navigating the patient through a complex process in the most efficient, effective, and acceptable way. There are many variants of case management within patient-centered and integrated care services; however, patient advocacy or the brokerage case management model is considered the most suitable.[6,7] The focus of the case management model is on a more comprehensive coordination of services across the continuum of care, as viewed from a patient perspective.[8] In this model, also referred to as the socioeconomic model, care coordination is determined not only by the patient’s medical needs but also by the financial, psychological, and social circumstances of the patient.[7,9]

Embrace is an example of a person-centered and integrated care service for community living older adults, in which case management based on the patient-advocacy model plays a central role.[10] Embrace is based on the Chronic Care Model (CCM) and a population health management model, the Kaiser Permanente triangle. The CCM is a model that integrates community social care and healthcare services, and has four interdependent key elements: self-management support, delivery system design, decision support, and clinical information systems.[11] The Kaiser Permanente triangle is used to segment the population of older adults, based on the self-reported levels of case complexity and frailty. Care coordination is performed by case managers who participate in a general practitioner-led multidisciplinary elderly care team, in which an elderly care physician also participates. The elderly care teams provide older adults with comprehensive, person-centered, proactive, and preventive care and support.[2,12]

Little is known about how redesigning healthcare delivery, especially in terms of case management, affects healthcare professionals. We therefore explored how case managers experienced their professional roles in a person-centered and integrated-care service for community-living older adults.

## Methods

### Design

We carried out a qualitative study using a grounded theory approach[13] to explore the experiences and personal perspectives of case managers. Data were collected by a trained interviewer (KS) through in-depth interviews, conducted 15 to 18 months after the Embrace program began in 2012. The methods were defined according to the Consolidated Criteria for Reporting Qualitative Research (COREQ) checklist.[14] Our study was carried out within the setting of a randomized controlled trial, focused on the effectiveness of person-centered and integrated care, the Embrace study.[10]

### Setting and intervention

Embrace requires an Elderly Care Team (ECT) within each of the 15 participating GP-practices located in the northern part of the Netherlands. For each participating GP-practice, a GP-led ECT was assembled, which consisted of an elderly care physician (also known as a nursing home physician), a community nurse (case manager for older adults with risk profile “Complex care needs”), and a social worker (case manager for older adults with risk profile “Frail”) in addition to the GP. The ECT had to provide comprehensive, proactive, preventive, person-centered care and support. The ECT-members aim is to elicit individuals’ values and preferences, and use them to guide all aspects of their health care, supporting their realistic health and life goals. The ECT seeks to establish dynamic relationships between the older adults, others who are important to them, and all relevant providers. This collaboration informs decision-making to the extent that the older adult desires. [12]

Participants within the profiles “Frail” and “Complex care needs” received individual care and support from a case manager, who acts according the patient-advocacy model for case management, that he or she responds to the needs and preferences of the older adult, rather than to the organization preferences. The case manager visited the older adults at home and focused on the older adults’ self-defined health-related problems such as mobility, physical health, mental health, and support from the environment, using an ICF-based assessment tool, the GeriatrICS.[15]

After the self-defined problems had been clarified the case manager and older adult prioritized problems and formulated goal plans using goal attainment scaling. During subsequent visits, the case manager monitored and navigated the older adult and evaluated and update the goal plans. Once every four weeks the ECT discussed the progress in the goal plans and the effectiveness of the interventions. Within one year, these goal plans were evaluated and an ‘obtained goal score’ was given, again by the older adult. Older adults within the profile “Robust” were monitored by the Elderly Care Team, which reviewed their medical files and medications at least once a year, while these older adults were invited to contact the ECT in case of emerging health-related problems. All participating older adults were offered a self-management support and prevention program with regular community meetings that emphasized preventive measures and endorsed a healthy lifestyle focusing on mobility, nutrition and social participation, while maintaining control and self-management abilities.

All ECT members complete a training program that focuses on their new professional roles. GPs follow a training program (3 days in total) related to, among other things, team and population management. The social workers (SW) and district nurses (DN) in the ECT followed an 8 days training program. They were trained in providing case management according to the principles of the patient advocacy case management model,[6] including proactive teamwork, preventive care, how to apply self-management support methods (such as motivational interviewing techniques), how to provide group-interventions to enhance the self-management ability and well-being for older adults, [16] and how to use the Electronic Elderly Record System (EERS). The EERS enables registration of care delivery, and contains goal plans that are reviewed during structured home visits (approximately once a month). All ECT members receive monthly on-the-job coaching during their team meetings.

Within Embrace, participating older adults were stratified into three risk profiles, based on self-reported case complexity (assessed with the INTERMED for the Elderly, self-assessment, INTERMED-E-SA) [17] and frailty (assessed with the Groningen Frailty Indicator, GFI).[18] The risk profiles were: “Robust” (INTERMED-E-SA score < 16 and GFI score < 5) for older adults without complex care needs and with a relatively low frailty level, “Frail” (INTERMED-E-SA score < 16 and GFI score ≥ 5) for frail older adults at risk for complex care needs, and “Complex care needs” (INTERMED-E-SA score ≥ 16) for older adults with complex care needs and at risk for institutionalization. Older adults with a “frail” profile received case management from the social worker, and older adults with “complex care needs” received case management from the district nurse (for detailed descriptions, see Spoorenberg et al.).[10]

### Study sample and data collection

All case managers received an invitation from one of the researchers (KS). Face-to-face in-depth interviews (average duration 107 minutes, range from 88 to 122 minutes) were held in the case manager’s office or another place of their choosing. Interviews were conducted by KS using a topic-based interview guide that consists of open-ended questions related to the key elements of the CCM and to case management in general (see S1 and S2 Files). After the first interview, the researchers (KS and SFM) evaluated the topic-based interview guide; only minor revisions were necessary. All interviews were audio recorded and transcribed verbatim. Transcriptions were reviewed for completeness and accuracy by KS.

### Data handling and analysis

We analyzed the data using a grounded theory approach[13], which is in line with current guidelines for qualitative data analysis.[19,20] We used Kwalitan 5.0 to support the data analysis.[21]

Analyses began with open coding of the first interview by two coders independently (KS and SFM). After the codes assigned were compared and discussed, an initial code list was established. For the next three interviews, the two coders coded the interviews independently, and then compared and discussed their findings. During this process, initial codes were preserved or revised, definitions modified, and new codes added. This resulted in a preliminary codebook. The two coders further revised this codebook during the analysis process by extending existing codes, shortening or eliminating redundant codes, or adding new codes.

After all the interviews were analyzed, the codes collected were clustered into categories. Major themes and subthemes were deduced and discussed by the researchers (KS, SFM, RU, KW) until consensus was reached. Next, quotes from the interviews were selected as illustrative and then returned to the participants to be checked for accuracy; the study findings were presented during a meeting with all the interviewees in order to facilitate feedback. The quotes and study findings were recognized and accepted by all the interviewees; only minor revisions were necessary such as the structure of the sentences and choice of words.

The analysis procedure was conducted in the source language (Dutch). An external, bilingual, certified translator translated the categories, themes, and quotations into English. The translations were reviewed by the researchers to ensure adequacy (see S1 Table for the original quotations in Dutch and their English translation).

### Ethical considerations

The Medical Ethical Committee of the University Medical Center Groningen assessed the Embrace study and concluded that their approval was not required (Reference METc2011.108). Informed consent from the case managers was obtained prior to the interviews, and transcripts were analyzed anonymously.

## Results

All eleven case managers (all female) participated in this study. Nine of them combined their case manager roles with their regular jobs as district nurses (DN) or social workers (SW) (see Table 1). We identified four major themes within the data, with a total of ten subthemes (see Table 2). The major themes were: 1) the changing relationship with older adults, 2) establishing the case manager role, 3) the case manager’s toolkit, and 4) the benefits of case management.

**Table 1:** Demographic and job characteristics of the case managers (n = 11).

Characteristics	Nurse case managers	Social worker case managers
N =	6	5
Age, year (mean, range)	52, 28–58	57, 49–61
Educational level (n, %)		
Intermediate vocational	-	1 (20)
Higher vocational	6 (100)	4 (80)
Job appointment (n, %)		
Embrace only	2 (33)	
Combination with regular job	4 (67)	5 (100)
Municipality (n, %)		
Stadskanaal	3 (50)	2 (40)
Veendam	2 (33)	2 (40)
Pekela	1 (17)	1 (20)

**Table 2:** Themes and subthemes identified.

Theme			Subtheme
1.	The changing relationship with older adults		Shift to person-centered approach
			Building a relationship of trust
2.	Establishing the case manager role		Case management
			Care and support plan
3.	The case manager’s toolkit		Knowledge and experience
			Competencies of case managers
			Preconditions for case managers
			Differences in professional background
4.	Benefits of case management		

### The changing relationship with older adults

The first theme relates to the change of focus that district nurses and social workers experienced in their new roles as case managers, “the shift to person-centered approach,” and the changes in their relationship with older adults as related to this new role, that is, “building a relationship of trust.” This can help the case manager to adopt the perspectives, values and preferences of the older adults in organizing care and support that addresses their physical, psychological, social and environmental health-related problems.

#### Shift to a person-centered approach

Case managers reported that their focus shifted from being a traditionally task-oriented one to one with a person-centered focus. This latter is based on a long-term relationship with the older adult that enables a case manager to gain an in-depth and broad view of the older adult’s needs and preferences, and to provide individualized and person-centered care and support.

*Since I adopted the perspective of the older adults themselves*, *I’ve been doing things their way, consistent with their own lifestyles and according to their own standards. (DN2)*

*I try to clarify what their concerns are, what is really bothering them, or find out what’s on their minds […]. Furthermore, you really take a look around, how their house is furnished […]. I often walk with them around the house […] and, if someone is 85 and can barely walk and has a huge garden, you’ll ask*, *“Gosh, how do you manage the garden?” Yes, it is all-inclusive […]. (SW2)*

#### Building a relationship of trust

All case managers mentioned the importance of establishing a relationship of trust with the older adult. Case managers invested in this relationship by taking sufficient time, being attentive, and by keeping their promises. A critical ingredient in all this was creating a sociable atmosphere, which was not a goal per se but rather a means of enhancing feelings of connectedness and of building a partnership.

*[…] it is important because it’s also a part of building a relationship of trust. Clients apparently like the social aspect, having a nice time*. *Well, I do think that this is an important component, but it’s certainly not my main reason for coming. (SW5)*

### Establishing the case manager role

This theme relates to the experiences of district nurses and social workers who provide case management in a person-centered and integrated-care service, and to the differences between district nurses and social workers as case managers.

#### Case management

The reflections of the case managers about providing case management relate to the central elements of person-centered and integrated care, such as proactive and preventive care delivery that includes monitoring, self-management support, care coordination, and network collaboration. Case managers stressed the need for a thorough knowledge of an older adult’s situation in order to make the early detection of problems feasible and to provide *proactive and preventive care*.

*[*…*] even if there aren’t any immediately obvious hazardous situations that could be expected*. *Keeping in contact could have a preventive effect*. (SW5)

*Monitoring* and reviewing the care situation of older adults, by means of regular home visits and follow-up by telephone, were regarded as essential and were figuratively described as “taking an older adults’ pulse.”

*You’re right there with the patients and following the processes*. *You start something up and then you monitor. If it’s not okay, you intervene. (DN1)*

Case managers indicated that they were able to adjust the intensity of their monitoring, based on the situation of the older adult. Some needed more frequent visits than average, while, for other older adults, a lower frequency of home visits in combination with contacts by telephone was more suitable. Case managers also indicated that they experienced a certain incompatibility between pro-active preventive care and Embrace guidelines regarding intensities of care and support provision. Embrace guidelines state that when goals have been attained and the situation has improved, older adults ought to be transferred to a less intensive care profile. Some case managers found this rather difficult, since they felt personally involved and responsible. In some situations they continued the case management after consultation with the Elderly Care Team.

Regarding *self-management support*, the aim of case managers was to increase older adults’ feeling of responsibility for their situation, to provide them with insight into their own abilities, to foster their sense of power, to motivate them to take steps on their own, and ultimately to take control of their own lives.

*A case manager is someone who maintains a critical overview of everything taking place with regard to the older person. They get things going, and they keep track, checking to see if things have actually been done, and whether they’ve been done properly. They really take a load off others’ shoulders, in my opinion. On the other hand, they are also able to encourage older adults to start doing things themselves. Just helping: “Yes, that’s right,” “You can do it yourself, like this,” and because people often have no idea how things work. And then they can do it themselves. It works both ways*: *being there for them and encouraging them to start doing things themselves. (DN1)*

Case managers felt responsible for *care coordination* but explicitly stated they were not responsible for care provision. They emphasized the importance of this independent position. Their activities were aimed at achieving continuity of care and enhancing the independent living of older adults.

*Yes, you have to learn […], so you are not inclined to take over everything yourself (laughs), so yes, that is in itself still a competence maybe we as nurses still need to work on […]. I think the most important thing is that you have an (in)formal network. That you know where you to find relevant information and what possibilities there are [for care and support] in your neighborhood. But in any case, that I […] know where I can get the information. (DN4) […] My starting point is always to support the autonomy and independence of the older adult as long as possible […] so that the older adult can continue to live independently at home for as long as possible. My position as a case manager, so to say, ‘at the service of the older adult’, and I act in his or her interest, and not in the interest of my discipline as nurse or organizations. Generally this is no problem*.*(DN4)*

Within the Elderly Care Team, case managers viewed themselves as representatives (“messengers”) of the older adults. The *collaboration* between the Elderly Care Team members was perceived as satisfactory. Sharing medical information and discussing physical functioning led to a broader perspective on older adults’ situations. Case managers experienced appreciation on the part of the GP; the GP, in turn, had confidence in them. Furthermore, case managers experienced better access to the GP, when it came to discussing the needs of the older adults.

#### Care and support plan

Case managers agreed that, although time-consuming, the computer-based individual care and support plan (part of the EERS) was a suitable method for gaining a complete overview of the situation of the older adult. Some case managers, however, experienced the use of a computer as a barrier in their communication with older adults.

*After a while, I would often decide not to bring the laptop along. Instead, I’d just take a look at the EERS in advance and update it afterwards. I think it interferes with the conversation. If you’re sitting there with a laptop while you’re having a conversation, I think it gets in the way somehow*. *(DN4)*

The comprehensive assessment of an older adults’ situation, mostly completed by the second or third home visit, provided a case manager with in-depth insight and helped to establish a basis for continuation of the care process. Case managers indicated, however, that older adults had difficulty judging the severity of their problems and had even more difficulty judging the feasibility of solving those problems. The same difficulties were experienced during the annual review of the care plan, in which older adults evaluated the past year’s achievements and set new goals.

### The case manager’s toolkit

Case management within a person-centered integrated care service requires, according to the interviewees, knowledge and experience, as well as competencies. Furthermore, several preconditions were found to be essential and case managers acknowledged differences in their professional backgrounds.

#### Knowledge and experience

All case managers agreed that higher vocational education (college-level education) was needed as well as basic medical knowledge regarding ageing and the consequences of ageing in order to provide case management. In addition, work experience in the care and support of older adults, and knowledge of local healthcare organizations and community services, was deemed essential.

*I think the most important thing is to have a good knowledge of the network–knowing where you can turn for things and what the possibilities in your district are. At any rate, so that I (…) know where I can find information. If even I don’t know, you can just imagine how difficult it would be for clients*. *(DN4)*

#### Competencies of case managers

Case managers listed the various competencies necessary for performing the case manager role, such as the ability to separate the essential from the non-essential, and to establish productive interactions with the older adults, the members of the Elderly Care Team, and other professionals.

Essential *communication and collaboration skills* were: listening and asking the right questions, understanding implicit messages, and providing feedback. Skill in motivating and stimulating older adults to improve their self-management abilities and independence were also considered just as important. *Observational skills* were deemed essential in order to detect existing problems and to anticipate future ones, as, for example, in observing the condition of the housing and living situation, as well as the physical appearance, personal hygiene, and the mobility of older adults. Furthermore, one case manager indicated that it was essential to be flexible and creative in terms of planning activities so that they were attuned to older adults’ daily schedules as well as to the varying number of their clients throughout the year.

*(…) you’re constantly having to adapt. You have to set aside your own values and beliefs and just observe how others live. This is very important. (…) you have to be open, avoid being judgmental. People live their own lives in their own way: Coffee gets reheated; they’ve been doing that their whole life*. *(…) I accept people for who they are; that’s something you have to learn. (DN1)*

#### Preconditions for case managers

Case managers listed several preconditions that enabled them to fulfill their roles, such as autonomy, a quiet workplace, training, and support from the Embrace project leaders. Most case managers reported a lack of support from the manager of their own organization and had trouble combining two jobs as case manager and community nurse or social worker They also expressed the need to swap experiences and to harmonize with fellow case managers because of the experimental nature of their role and the uncertainty about how to fulfill it.

*It’s still in the development stage, and you need feedback. Now and then, you need to be able to talk it out with colleagues to see if you’re on the right track*. *(DN1)*

*No, I do not prefer to combine two jobs. I find it very difficult, especially switching. […] If you have two jobs and you have certain responsibilities, you do not say 'Okay, the 16 hours are over’*. *(SW3)*

#### Differences in professional background

Case managers with a background in social work (SW) felt that they focused more on the psychosocial aspects, such as disturbed interpersonal relationships and loneliness, as compared to district nurse (DN) case managers. In contrast, DN case managers felt that they focused more on solving healthcare and medical problems than SW case managers. DN case managers considered that some SWs had insufficient medical knowledge and were therefore unable to detect older adults’ physical problems, thus possibly hindering the provision of preventive care. Nevertheless, this difference in expertise might also prove to be an advantage in the delivery of care:

*I once was visiting a couple and (…) they were having trouble filling in a questionnaire. I thought that the questions were too difficult for them, and I just couldn’t understand. And they were having some problems with their house, and they weren’t getting anywhere with them. So I told [name of the social worker], “I just don’t know what I should do.” [Name of the social worker] went over there for an hour. Neither of these people could read and write properly, and they were having a real hassle with the housing corporation. No wonder–they couldn’t read the housing corporation’s forms. [Name of the social worker] was able to get this out in the open with her approach and manner of questioning*. *(DN1)*

Case management closely follows the framework of the nursing process, and so it was therefore a new method of working for case managers with an SW background. In addition, SW case managers felt their lack of experience when participating in “medical meetings” and in verbalizing medical problems concisely. During Elderly Care Team meetings, they at first felt restricted to introducing topics that were related to well-being.

### Benefits of case management

For most case managers, their new role was truly satisfying and had transformed their career perspectives. The satisfaction of older adults was regarded as highly rewarding. One case manager stated she had felt less satisfied at first, since it seemed to her she was not able to “actually do” anything, but later she realized that attentive listening, for example, was also “doing something.” The role of case manager provided case managers with a new outlook on their regular jobs as SWs or DNs.

Case managers felt that older adults perceived them as confidants and gladly shared personal information with them. They had the feeling that older adults appreciated the personal attention, which gave these older adults a sense of strength and support.

*(…) after I’d been there as a case manager, he felt stronger. With the tips he received, he was able to start enjoying life again and doing some things on his own*. *(DN2)*

Case managers, acting as “brokers,” felt that they were truly able to make a difference by organizing care and support in collaboration with other organizations or professionals.

*She [an older woman with physical and psychological symptoms] was receiving home care, and all kinds of organizations dropped by, but it didn’t seem as if anyone really had a proper understanding of what was going on*. *(DN6)*

The case managers were modest about their contribution to older adults and often labeled their activities as “small acts,” while at the same time stressing the importance of those seemingly small acts for older adults:

*When I started with Embrace, I knew that small things could make a world of difference to older adults. Having a good wheelchair or not having a good one makes a huge difference*. *(DN1)*

## Discussion

The aim of this study was to explore how case managers experience their professional role in a person-centered and integrated care service for community-living older adults. We found that this major role change was experienced as a learning process, providing opportunities for personal and professional growth. Case managers felt that they were able to make a difference, and found their new role truly satisfying and challenging, albeit stressful at times as well. Ongoing training and support was found to be a prerequisite for facilitating the shift in focus toward person-centered and integrated care.

Case managers indicated that their relationship with older adults had changed. Trust was found to be essential in order to be able to establish their case-manager role, which confirmed findings from the few previous qualitative studies on case management for older adults.[22,23] We also found that the boundaries of the traditional patient-professional relationship were often crossed, transforming the relationship into a hybrid form of informal and formal service-oriented relationship.[24] The focus of such a relationship is to empower and help older adults remain in control of their lives,[25] as well as to improve patient outcomes.[26,27] A relationship that is built on trust should therefore be seen as a prerequisite for acting as an older adult’s representative or “voice” when organizing and coordinating care and support.

We found that case managers were successful in establishing the case manager role and that they had also convincingly adopted the patient advocacy model. The regular home visits enabled case managers to take an older adult’s “pulse”, and enabled them to coordinate care with the aim of enhancing the independent living of the older adult rather than having to provide the care themselves. Although they were not responsible for care provided by others, they did feel responsible at times. Especially when they combined their role as case manager with a job as a community nurse or social worker. This is in line with findings of similar qualitative studies, in which case managers of frail older adults described themselves as “coaching guards,” alluding by this to their underlying roles as problem-solvers, supporters, sentinels, and navigators.[22,23] Case managers were also able to focus on the financial, psychological, and social circumstances of the older adult; this is essential in order to provide person-centered and integrated care.

Regarding the case manager’s toolkit, we found that a case manager’s knowledge and experience, combined with specific competencies, were essential for fulfilling the role. This is in line with our findings from a longitudinal study on the levels of implementation of integrated care.[28] Furthermore, we found similar differences between the two types of case managers, as Park and colleagues had.[29] Nurses tended to focus on health conditions, while social workers addressed psychosocial and financial problems. However, during the Elderly Care Team meetings and home visits, case managers were able to exchange information and knowledge. So these differences in professional expertise not only served to benefit the delivery of care but also their own professional development.

We found that not all the preconditions needed for case management were being met, however. Challenges in daily work, such as a high caseload, combining the case-manager role with a regular job, as well as case closures led, on some occasions, to stress and role conflict, often referred to at times as a challenge to professional identity.[23] This also, in turn, could interfere with job satisfaction.[30] Case managers, moreover, sometimes expressed feelings of isolation and uncertainty about how they were functioning.[31] In addition, case managers indicated that Embrace guidelines sometimes hampered the execution of their role, especially regarding the continuation of their professional relationship with older adults. It is therefore important to involve case managers in revisions and further development of guidelines and organization of case management as well as to involve their managers. Finally, it is important to provide continuous training and support, including peer-to-peer coaching.

The strengths of this study lie in researcher triangulation (two coders), using a code book, constant comparison, keeping field notes (memos), and intercoder discussion, which increased validity and reliability. Regarding the validity of the data interpretation, feedback on study findings was obtained from project leaders and researchers from the Embrace study. Some limitations need to be noted as well, however. The small sample size might be seen as a potential limitation in terms of data saturation. Although all Embrace case managers participated in this qualitative study, further research is needed to confirm the results found.

## Conclusion

We found that the case managers experienced providing person-centered and integrated care as being very rewarding, but it required a major role change for them. They experienced this as a learning process that provided opportunities for personal and professional growth. Research regarding the effectiveness of Embrace showed that quality of care improved, [32] that Embrace reinforced older adults’ ability to remain in control and that they felt safe and secure, which is in contrast to the fears of increasing dependency within the usual care system.[33] A study on the change in the prevalence and severity of health-related problems showed that case management within Embrace offers a route to counteract the decline in physical, cognitive and social functioning associated with ageing.[34] On the other hand, after 12 months of its use, Embrace was not yet demonstrably to be cost-effective [35] and had no clear beneficial effect on patient-reported health status and neither on wellbeing and self-management outcomes. [36] At the moment, the balance of these various effects has still to be made.

## Supporting information

S1 File Interview guide (Dutch original). (DOCX)

S2 File Interview guide (English). (DOCX)

S1 Table Original quotations in Dutch with English translation. (DOCX)
