# Supplementary material for: Experiences of case managers in providing person-centered and integrated care based on the Chronic Care Model: A qualitative study on embrace
Source: PLoS One. 2018 Nov 15;13(11):e0207109. doi: 10.1371/journal.pone.0207109 (PMC6237343; doi:10.1371/journal.pone.0207109)
Supplement: S1 File — (DOCX) [file pone.0207109.s001.docx]

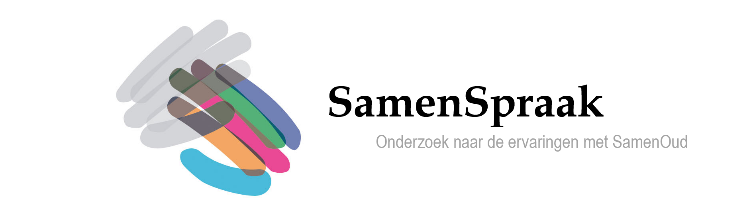


**Interviewschema SamenSpraak 2, versie 12 mei 2013**

**Introductie**

- Doel: We willen graag jouw ervaringen en mening horen over de rol en de essentie van de casemanager binnen SamenOud.
- Presentje geven: iets bij de koffie
- **Anonimiteit garanderen**
  - Uitleg + tekenen toestemmingsformulier toestemmingsverklaring

**Interviewvragen**

Persoonlijke vragen: werkervaring, wv / oa?, opleiding, geb. datum. ouderenzorg

1. Wat is SamenOud voor jou in één zin?

*Rol/functie casemanager*

1. Wat houdt casemanagement voor jou in? Is er verschil tussen casemanagement en casemanager?
2. Wat is de rol van de casemanager binnen SamenOud?
3. Wat houdt de rol volgens jou in? Hoe vul jij deze rol/ functie in?
4. Wanneer ben je een goede cm? Wanneer werkt cm niet?
5. Wat is er veranderd vergeleken met als je niet volgens SamenOud werkt?
6. Wat is volgens jou kenmerkend aan de SamenOud casemanager?
7. Wat vind je belangrijk binnen je rol?
8. Wat is je verantwoordelijkheid als casemanager?
9. Wat vind jezelf de meerwaarde van jouw rol als casemanager t.o.v. als je niet volgens SamenOud werkt? Komt deze tot zijn recht? Hoe?
10. Hoe zie je jouw plek t.o.v. de oudere (positie)?
11. Hoe kan je zorgen voor meer **eigen regie**, zelfredzaamheid, veiligheid en welbevinden voor een oudere? Voorbeelden!
12. Hoe zijn je ervaringen met de zorg en begeleiding die je nu geeft in het kader van SamenOud?
13. Welke ervaringen heb je t.a.v.:

- Anamnese: **wonen, welzijn en zorg**
- Opstellen Zorgleefplan
- Organiseren van zorg en begeleiding (monitoren, navigeren naar andere hulpverleners)
- Regelmatig contact houden met oudere
- Evaluatie (klein, groot)

1. Wat waren je verwachtingen van je functie als SamenOud casemanager? Komen deze uit?

*Resultaten / opbrengsten*

1. Wat heb je in jouw rol als cm voor de ouderen kunnen betekenen / bereiken? Voorbeelden?
2. Wat heeft het jou opgeleverd?
3. Wat vind je belangrijk om te bereiken?

*Ouderenzorg Team (OT)*

1. Wat is je rol binnen het OT?
2. Heb je deze rol ook goed kunnen vervullen? Hoe?
3. In welke mate vertegenwoordig je een oudere binnen het OT?
4. Hoe realiseer je wat je wilt binnen het OT?
5. Heb je verbeterpunten voor het OT? Welke?

*Nodig?*

1. Wat heb je nodig om je functie goed uit te oefenen? In welke mate was dat aanwezig?
2. En als je denkt aan:
   1. Opleiding / bijscholing (welke nodig?)
   2. Ervaring?
   3. Welke ondersteuning vanuit OT, eigen management, projectteam?
   4. Welke gedragskenmerken /eisen?

- Managen complexe processen, Netwerken *(kunnen netwerken/ hebben van netwerk)*, Samenwerken, Pionieren / vernieuwend, Pro-actief, Kan werken met digitale systemen, Plannen
  - - - Zelfmanagement ondersteuning, Ontwerp van het zorgproces, Besluitvormingsondersteuning, Klinische informatie systemen, Voorbereid proactief team, Geïnformeerde geactiveerde cliënt, Productieve interacties, Plek binnen de organisatie(s)]
  1. Uren?
  2. Werkplek?
  3. ICT (EOD)?
  4. Type aanstelling (fulltime/parttime)
  5. Overlegvormen (Ouderenzorg Team, casemanagersoverleg)
  6. Welke richtlijnen / regels etc?

*Verbeterpunten / knelpunten?*

1. Wat liep er lekker het eerste jaar van SamenOud? Wat liep er niet lekker het eerste jaar van SamenOud?
2. Hoe kan SamenOud volgens jou nog verbeterd worden?
3. Toekomst: zijn de 'opbrengsten' voor ouderen zo groot is dat SamenOud voortgezet moet worden? Hoe zou de toekomst van SamenOud er uit moeten zien?

Reminders

- Willen, kennen, kunnen, mogen
