# Supplementary material for: Experiences of case managers in providing person-centered and integrated care based on the Chronic Care Model: A qualitative study on embrace
Source: PLoS One. 2018 Nov 15;13(11):e0207109. doi: 10.1371/journal.pone.0207109 (PMC6237343; doi:10.1371/journal.pone.0207109)
Supplement: S2 File — (DOCX) [file pone.0207109.s002.docx]

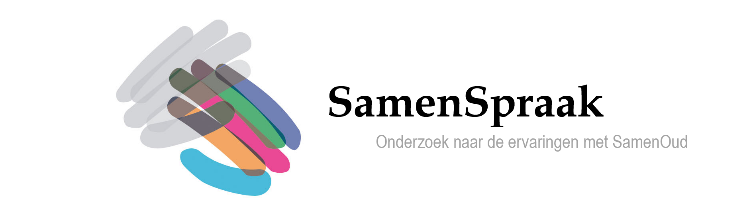


**Interview guide Samenspraak 2**, version 12 May 2013

**Introduction**

• Goal: We would like to hear your experiences and opinions and essence of the role of case manager

• Give a present: something with the coffee

• Guarantee anonymity

• Explanation + sign permission

**Interview questions**

Personal questions: work experience, nurse, social worker, education, birthdate, elderly care team

1. What is Embrace for you in one sentence?

**Role / function of case manager**

2. What does case management mean to you? Is there a difference between case management and case manager?

3. What is the role of the case manager within Embrace?

a) What does the role mean to you? How do you complete this role / function?

b) When are you a good case manager? When doesn’t case management work?

c) What has changed compared to if you do not work according to Embrace?

d) What do you think is typical for the Embrace case manager?

e) What do you find important in your role?

f) What is your responsibility as a case manager?

g) What do you consider to be the added value of your role as a case manager compared to if you do not work according to Embrace? Does this work? How?

h) How do you see your place in relation to the older adult (position)?

i) How can you provide for more self-management, self-reliance, safety and well-being for an older adult? Examples!

4. How are your experiences with the care and support you provide in the context of Embrace?

a) What experiences do you have regarding.:

• history taking: living, wellbeing and care

• Drafting Care Plan

• Organizing care and support (monitoring, navigating to other emergency services)

• Keep regular contact with older adults

• Evaluation (small, large)

5. What were your expectations for your role/ position as Embrace case manager? Did they come true?

**Results / revenues**

6. What have you been able to mean / achieve in your role as cm for the elderly? Examples?

7. What did it bring you?

8. What do you find important to achieve?

**Elderly Care Team (OT)**

9. What is your role within the OT?

a) Have you been able to fulfill this role well? How?

b) To what extent do you represent an elder within the OT?

c) How do you realize what you want within the OT?

d) Do you have any improvement points for the OT? Which?

**Required?**

10. What do you need to properly exercise your job? To what extent was that present?

11. And if you think of:

a) Training / further training (which need?)

b) Experience?

c) What support from elderly care team, your management, project team?

d) What behavioral characteristics / requirements?

Managing complex processes, Networks (can network / have network), Collaborate, Pioneer / innovative, Pro-active, Can work with digital systems, Plans

Self-management support, Design of the care process, Decision-making support, Clinical information systems, Prepared proactive team, Informed activated client, Productive interactions, Place within the organization (s)]

e) Hours?

f) Workplace?

g) ICT (EERS)?

h) Type of appointment (full-time / part-time)

i) Consultation forms (Elderly Care Team, case managers consultation)

j) Which guidelines / rules etc?

**Points for improvement / bottlenecks?**

12. What went well with the first year of Embrace? What was not going well for the first year of Embrace?

13. According to you, how can Embrace be improved?

14. The future: are the 'revenues' for older people so great that Embrace needs to be continued? What should the future of Embrace look like?

**Reminders**

• Wanting, knowing, being able, allowed
