# Supplementary material for: Experiences of case managers in providing person-centered and integrated care based on the Chronic Care Model: A qualitative study on embrace
Source: PLoS One. 2018 Nov 15;13(11):e0207109. doi: 10.1371/journal.pone.0207109 (PMC6237343; doi:10.1371/journal.pone.0207109)
Supplement: S1 Table — (DOCX) [file pone.0207109.s003.docx]

**Supplementary Table S1** Original quotations in Dutch with English translation

| **Dutch** | **English** |
| --- | --- |
| “Je staat echt naast die patiënten en je volgt processen. Je zet iets in gang en je volgt het. Als het niet goed is, grijp je in.” (DN1) | “You’re right there with the patients and following the processes. You start something up and then you monitor. If it’s not okay, you intervene.” (DN1) |
| “[...] ook al is er niet echt heel duidelijk een calamiteit te verwacht. Juist dan kan het preventief werken om wel contact te houden.”(SW5) | “[...] even if there aren’t any immediately obvious hazardous situations that could be expected. Keeping in contact could have a preventive effect.” (SW5) |
| Een casemanager is iemand die alles rondom de oudere volgt en ook kritisch volgt.  Die dingen in werking zet en het blijft volgen.  Die gaat kijken of dingen echt zijn gebeurd of dingen goed zijn gebeurd.  Die anderen echt een zorg van de schouders neemt vind ik.  En aan de andere kant ook wel weer iemand die ook de oudere kan prikkelen om weer dingen zelf te ondernemen.  Even weer helpen ‘oh ja zo was het’, ‘U kan het zelf, het werkt zo..’, want vaak hebben mensen ook geen idee hoe dingen moeten.  Dan kunnen ze het zelf.  Dus het is twee kanten, én er naast staan, maar ook prikkelen om dingen weer zelf te ondernemen.” (DN1) | “A case manager is someone who maintains a critical overview of everything taking place with regard to the older person. They get things going, and they keep track, checking to see if things have actually been done, and whether they’ve been done properly.  They really take a load off others’ shoulders, in my opinion.  On the other hand, they are also able to encourage older adults to start doing things themselves.  Just helping, ‘Yes, that’s right,’ ‘You can do it yourself, like this,’ and because people often have no idea how things work.  And then they can do it themselves.  It works both ways: being there for them and encouraging them to start doing things themselves.” (DN1) |
| “Heb ik toch op een gegeven moment ook vaak voor gekozen om niet de laptop mee te nemen en gewoon vooraf in het EOD te kijken en ook achteraf in het EOD weer bij te werken.  Ik vind ‘m wel belemmerend in een gesprek.  Ik vind als jij een laptop op schoot hebt en je zit, je bent in gesprek met iemand, ik vind ‘m toch een soort van belemmering.” (DN4) | “After a while, I would often decide not to bring the laptop along. Instead, I’d just take a look at the EERS in advance and update it afterwards.  I think it interferes with the conversation.  If you’re sitting there with a laptop while you’re having a conversation, I think it gets in the way somehow.” (DN4) |
| “Ik was bij een echtpaar en […] die hadden moeite een vragenlijst in te vullen. En toen dacht ik dat die vragen te moeilijk waren, en ik begreep het gewoon niet. En er waren wat problemen met hun woning en dat schoot maar niet op. Dus toen zei ik tegen [naam ouderenadviseur], ik zeg: ‘Nou [naam ouderenadviseur] ik weet gewoon niet wat [ik hier mee moet]...’. [naam ouderenadviseur], was er een uur geweest. Die mensen konden dus alle twee niet goed lezen en schrijven, hadden een heel gedoe met de woningbouw. Ook geen wonder want ze konden de formulieren van de woningbouw dus ook niet lezen. Dus [naam ouderenadviseur] had door haar manier van vragen en benaderen dat boven tafel.” (DN1) | “I once was visiting a couple and (…) they were having trouble filling in a questionnaire. I thought that the questions were too difficult for them, and I just couldn’t understand. And they were having some problems with their house, and they weren’t getting anywhere with them. So I told [name of the social worker], ‘I just don’t know [what I should do]...’ [Name of the social worker] went over there for an hour. Neither of these people could read and write properly, and they were having a real hassle with the housing corporation. No wonder – they couldn’t read the housing corporation’s forms. [Name of the social worker] was able to get this out in the open with her approach and manner of questioning.” (DN1) |
| Sinds ik vanuit de oudere kijk dan doe ik het op de manier zoals zij dat willen. Binnen hun levensstijl en maatstaven.” (DN2) | “Since I adopted the perspective of the older adults themselves, I’ve been doing things their way, consistent with their own lifestyles, and according to their own standards.” (DN2) |
| “[…] probeer ik verheldering te krijgen waar hun zorgen liggen, wat hun bezighoudt, wat hun dwars zit. […]  En daarnaast kijk je heel erg, hoe is het ingericht […] gewoon praktisch, qua veiligheid, losse matjes, wc‘s aanpassingen, douche aanpassingen. Vaak loop ik ook met de mensen […] even door het huis […]. Kijk, en als iemand een enorme tuin heeft en die is 85 en die kan amper lopen, ja, dan vraag je toch, ‘Goh, hoe doet u dat met de tuin?‘ Ja, dat neem je allemaal mee […].” (SW2) | I try to clarify what their concerns are, what is really bothering them or find out what’s on their minds [...]. Furthermore, you really take a look around, how their house is furnished [...] I often walk with them around the house [...], and if someone is 85 and can barely walk and has a huge garden, you’ll ask, ‘Gosh, how do you manage the garden?’ Yes, it is all inclusive” [...]. (SW2) |
| “[…] het is belangrijk omdat 't ook een onderdeel is van het kweken van een vertrouwensband. Gezelligheid vinden de cliënten zelf kennelijk plezierig. Nou, ik vind het een belangrijk element, maar het is natuurlijk niet waarvoor ik echt kom.” (SW5) | “[…] it is important because it’s also a part of building a relationship of trust. Clients apparently like the social aspect, having a nice time. Well, I do think that this is an important component, but it’s certainly not my main reason for coming.” (SW5) |
| “Ja, dat moet je dus leren, dan moet je dus zelf niet zo geneigd zijn om alles over te nemen (lacht), dus ja, dat is op zich nog wel een kunst ja, die wij misschien als wijkverpleegkundigen juist niet zo heel erg bezitten, omdat wij geneigd zijn om dingen te regelen voor anderen […] Ik denk dat het belangrijkste is dat je het netwerk goed kent. Dat je heel goed weet waar je terecht kunt voor dingen en wat er dus allemaal in je wijk voor mogelijkheden zijn. Maar in ieder geval en dat ik […] weet waar ik de informatie vandaan haal […] Mijn uitgangspunt is steeds de autonomie en onafhankelijkheid van de oudere zo lang als mogelijk en wenselijk is te ondersteunen, zodat de oudere zolang mogelijk zelfstandig thuis kan blijven wonen. Ik als casemanager sta ten dienste van de oudere, zo gezegd, en handel in diens belang, waarbij ik discipline- en organisatie overstijgend denk en handel. In algemeen is dit ook geen probleem. (DN4)” | Yes, you have to learn […], so you are not inclined to take over everything yourself (laughs), so yes, that is in itself still a competence maybe we as nurses still need to work on […]. I think the most important thing is that you have an (in)formal network. That you know where you to find relevant information and what possibilities there are [for care and support] in your neighborhood. But in any case, that I [...] know where I can get the information. (DN4)  […] My starting point is always to support the autonomy and independence of the older adult as long as possible [...] so that the older adult can continue to live independently at home for as long as possible. My position as a case manager, so to say, ‘at the service of the older adult’, and I act in his or her interest, and not in the interest of amy discipline as nurse or organizations. Generally this is no problem.(DN4) |
| “[…] dus je moet je overal aanpassen.  Je eigen normen en waarden moet je wegduwen en je moet gewoon kijken naar hoe anderen leven.  En dat is dus gewoon heel belangrijk. […] je moet open staan. Niet oordelen, niet veroordelen. Mensen leven hun eigen leven zoals ze leven, koffie wordt opgewarmd, dat zijn ze hun hele leven gewend. […] Ik laat mensen in hun waarde, dat moet je leren.” (DN1) | “(…) you’re constantly having to adapt. You have to set aside your own values and beliefs, and just observe how others live.  This is very important. (…) you have to be open, avoid being judgmental.  People live their own lives in their own way: coffee gets reheated; they’ve been doing that their whole life. (…) I accept people for who they are; that’s something you have to learn.” (DN1) |
| Het is nog steeds in ontwikkeling en je hebt feedback nodig. Je moet af en toe kunnen sparren met collega’s om te zien of je goed bezig bent.” (DN1) | “It’s still in the development stage, and you need feedback. Now and then, you need to be able to talk it out with colleagues to see if you’re on the right track.” (DN1) |
| “Geen tweede baan ernaast. Ik vind het heel moeilijk, ook het switchen. [...] Je hebt twee banen en die heb je. En als je bepaalde verantwoordelijkheid hebt dan zeg je niet 'Oké, de 16 uren zitten erop, nee” (SW3) | No, I do not prefer to combine two jobs. I find it very difficult, especially switching. [...] If you have two jobs and you have certain responsibilities, you do not say 'Okay, the 16 hours are over’. (SW3) |
| “(...) doordat ik nu geweest ben als casemanager daar, voelde die zich sterker. Hij had weer meer houvast in het leven omdat hij weer meer handvatten kreeg om weer zin het leven te krijgen en om dingen te ondernemen.” (DN2) | “(...) after I’d been there as a case manager, he felt stronger. With the tips he received, he was able to start enjoying life again and doing some things on his own.” (DN2) |
| “Zij had wel thuiszorg en allerlei instanties kwamen er ook hoor, maar niemand pikte de dingen echt op leek het wel.” (DN6) | “She was receiving home care, and all kinds of organizations dropped by, but it didn’t seem as if anyone really had a proper understanding of what was going on.” (DN6) |
| “Ik wist dus toen ik begon in SamenOud, dat voor de ouderen kleine dingen een wereld van verschil maken, ja. Wél een goede rolstoel of geen goede rolstoel maakt een heleboel verschil.” (DN1) | “When I started with Embrace, I knew that small things could make a world of difference to older adults. Having a good wheelchair or not having a good one makes a huge difference.”(DN1) |
